# Climatological Atlas of Temperature and Salinity for the Northeast Asian Seas

**DOI:** 10.1038/s41597-025-04543-6

**Published:** 2025-02-04

**Authors:** Yong Sun Kim, Soo-Hyun Seok, Jae-Ho Lee, Sung-Dae Kim

**Affiliations:** 1 Ocean Circulation and Climate Research Department, Korea Institute of Ocean Science and Technology, Busan, 49111 South Korea; 2 Department of Oceanography, University of Science and Technology, Daejeon, 34113 South Korea; 3 Ocean Science and Technology School, Korea Maritime and Ocean University, 49112 Busan, South Korea; 4 Climate Services and Research Division, APEC Climate Center, 48058 Busan, South Korea

**Keywords:** Physical oceanography, Physical oceanography

## Abstract

This study describes a monthly Atlas for the Northeast Asian Seas 2023 (ANAS23) with a 1/10° horizontal resolution and 73 vertical levels. For ANAS23, over 1.6 million hydrographic profiles were analyzed, utilizing a simple kriging interpolation technique, which considers data density and their covariance at each grid point, along with a profile stabilizing method to minimize damage to water-mass structures. Comparison of ANAS23 with previously published atlases, repeated sectional observations, and satellite-based geostrophic current fields reveals that the ANAS23 provides reliable descriptions of the spatial distribution of water masses, currents, thermohaline fronts, and mesoscale eddies while avoiding spike-shape noises, vertical instabilities, and artificial waters, particularly over large-topographic features. The ANAS23 could be utilized as a baseline to assess the dynamic state of climatological mean fields and their changes under evolving climates. The fact that uncertainty among atlases is still apparent, particularly in a region of scarce observations, calls for a collaborative international effort to collect qualified hydrographic observations for a better-performing regional atlas, thus improving predictive skills for future climate.

## Background & Summary

The gridded, climatological, objectively analyzed atlas provides a baseline for understanding the historical mean state of the ocean and assessing its temporal departures from the baseline under global warming. The fact that many numerical simulations take advantage of the gridded atlas to simulate historical as well as future climates by prescribing it as an initial condition demonstrates that skill for historical experiments and future projections might rely on the atlas’ realization of thermodynamic ocean structures. From the recognition of the climatological atlas’ implications on ocean and climate communities, the Levitus group at the National Ocean and Atmosphere Administration (NOAA) developed and published the World Ocean Atlas (WOA) in 1982. This pioneering global ocean climatology was constructed based on extensive *in*-*situ* observations accumulated over several decades^1^. As more *in-situ* observations became available, this group has periodically updated the WOA, becoming a prototypical and most widely used climatology^2,3^. Besides the WOA series, a few studies have introduced global atlases generated from increased observations from various global networks and using a cutting-edge methodology for quality control procedures and objective interpolation techniques^4–7^.

These global atlases focus on large-scale mean features, adopting a relatively large influence radius (or correlation length) for interpolation and noise-filtering procedures^2,6–8^. This approach efficiently suppresses high-frequency noises and allows for producing a climatological mean field; it also obscures mesoscale features, which are essential to understanding thermodynamic structures in a marginal sea^9–11^. Moreover, this radius could forge warmer and saltier waters at grid points close to the seafloor biased to the observed data in the open ocean, particularly in areas with a large-scale topographic feature like continental slopes, resulting in an artificial water mass and often invoking vertical instabilities^12^. A robust stabilization procedure, therefore, should be employed to address these instabilities. This artificial process could, in turn, potentially introduce wiggles in temperature and salinity profiles^5^. These issues reflect that the global atlas with a large influence radius might be inappropriate for in-depth studies on marginal seas^9–15^.

**Fig. 1:**
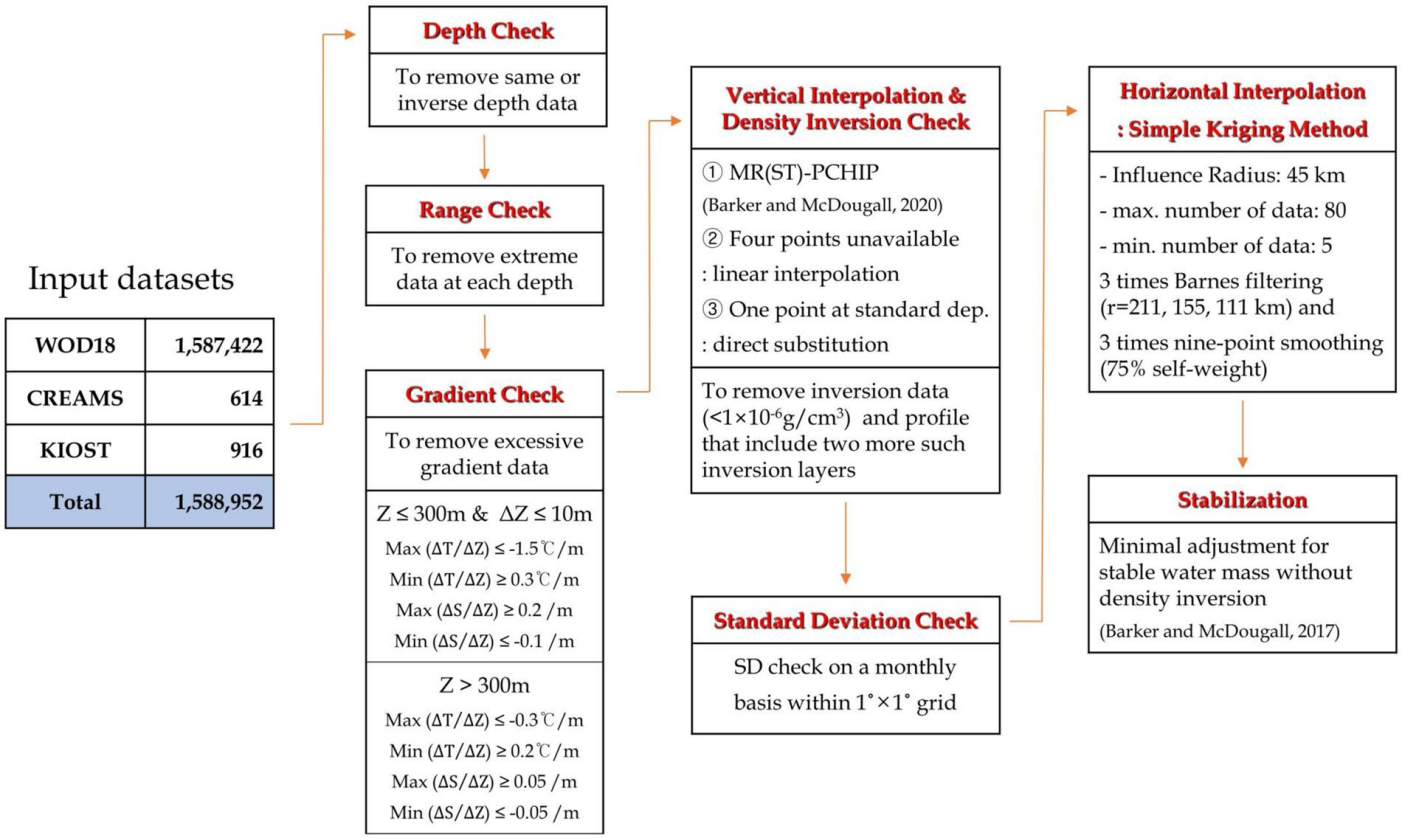
Flowchart outlining the production process of the ANAS23.

**Fig. 2:**
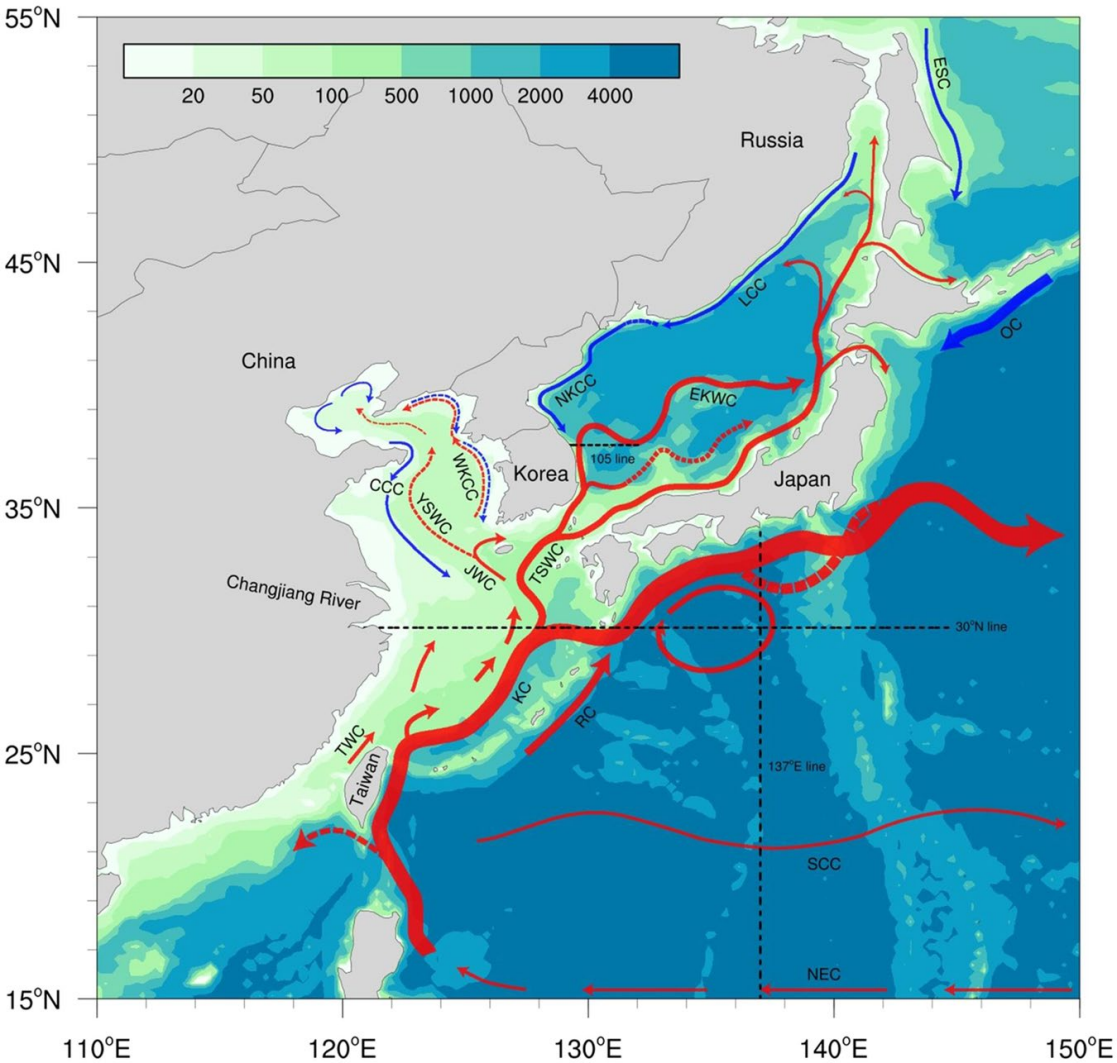
The ocean currents (the red and blue arrows representing warm and cold currents, respectively) and bathymetry (shaded, m) of the East Asian Seas. The dashed arrows indicate currents with high spatial or temporal variability. Abbreviations are CCC, Chinese Coastal Current; EKWC, East Korea Warm Current; ESC, East Sakhalin Current; JWC, Jeju Warm Current; KC, Kuroshio Current; LCC, Liman Cold Current; NEC, North Equatorial Current; NKCC, North Korea Cold Current; OC, Oyashio Current; RC, Ryukyu Current; SCC, Subtropical Counter Current; TSWC, Tsushima Warm Current; TWC, Taiwan Warm Current; WKCC, West Korea Coastal Current; YSWC, Yellow Sea Warm Current. The dashed lines represent verification lines.

This study aims to suggest a new approach to construct a regional climatological atlas that preserves mesoscale thermodynamic features and water masses in a marginal sea (Fig. 1, https://www.khoa.go.kr/oceangrid/gis/category/observe/observeSearch.do?type = EYS#none). To address this approach, we have focused on the marginal seas in East Asia, which includes the Bohai Sea, the Yellow and East China Seas (YECS), and the East Sea (referred to as the Japan Sea) in the northwestern Pacific (Fig. 2). As a part of the East Asian monsoon system, these seas have experienced abrupt warming during the past several decades; concurrently, extreme events have occurred more frequently in recent years^16–24^. Earth system models have projected this tendency further to accelerate with ongoing global warming^25,26^. These climate changes invoke massive socio-economic damage to this large marine ecosystem, as well as people in high-density population countries around these seas^16,21,27^. Furthermore, the local changes in these seas, in turn, could impact the downstream regions throughout the northern hemisphere associated with the mid-latitude jet, eddy vorticity flux, and circum-global teleconnection^28–30^. Therefore, a thermodynamic modulation of these marginal seas in the Northwest Pacific to global warming has become an urgent global issue.

These seas amplified, localized responses to global warming could be attributed to complicated oceanic features associated with oceanic currents and discharged waters from the rivers around these seas^16,19,21,30,31^. Of particular significance is the Kuroshio, the western boundary current in the North Pacific, which principally determines the circulation of mass, heat content, and salt in our study area^32–35^. Its local branches into the East Sea via the East China Sea generate quasi-persistent eddies in the East Sea^36–38^, a subpolar front^39^, and the salinity-minimum intermediate cold water mass flowing from the north to the south along the eastern coast of Korea^37,40^. Besides the current system, the riverine waters from the Yangtze River, one of the world’s largest rivers, contribute to the amplification of atmospheric forcing by forming a barrier layer vertically^16,19,30^. The Yellow Sea Warm Current (YSWC), which is driven by northerly winds during the winter monsoon, results in a strong thermohaline front^41,42^.

**Fig. 3:**
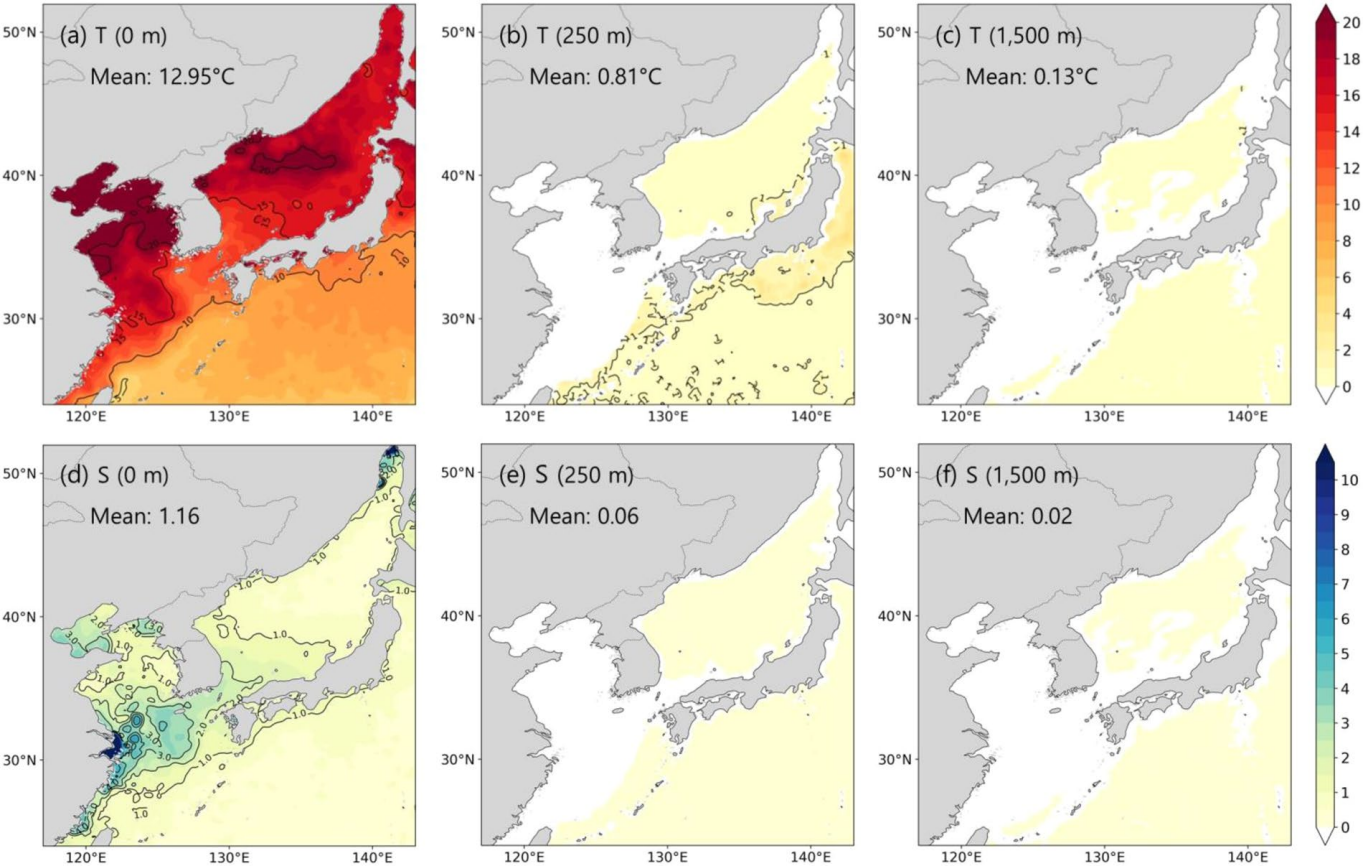
The spatial distribution of seasonal amplitudes for (**a**,**b**,**c**) temperature and (**d**,**e**,**f**) salinity at 0 m, 250 m, 1,500 m, respectively.

In this study, we developed a monthly regional Atlas for the Northeast Asian Seas 2023 (ANAS23)^43^ by adopting a simple kriging interpolation technique that considers data density and covariance for each grid point, along with a profile stabilizing adjustment^44^ after horizontal interpolation. The ANAS23^43^ has a horizontal resolution of 0.1° × 0.1° with 73 vertical levels from 0 to 5,500 m. The monthly mean climatology covers the upper 57 levels up to 1,500 m. The path along the Kuroshio Current^32^ and the areas with North Pacific Intermediate Water (NPIW)^45^, which is characterized by a salinity minimum layer (S < 34.1) centered around 700 m in the western North Pacific, showed weak seasonal variability in layers shallower than 1,000 m. However, at a depth of 1,500 m, the seasonal amplitude of temperature averaged 0.13 °C, and that of salinity averaged 0.02, which might be trivial compared to the ones at the upper layers (Fig. 3). Therefore, data below a depth of 1,500 m are provided as annual mean^6,46^. The construction process of ANAS23 is elaborated in the following sections. Its validation was assessed in terms of the horizontal and vertical distribution, temperature–salinity diagram, and geostrophic current by comparing ANAS23 with long-term serial hydrographic observations in the East Sea and south of Japan, satellite altimetry data and other gridded atlases with the same 1/10° and 1/4° horizontal resolution; these atlases include East Asian Seas Regional Climatology (EAS-RC^14,47^; https://www.ncei.noaa.gov/products/east-asian-seas-regional-climatology), WOA18^48^ (https://www.ncei.noaa.gov/access/world-ocean-atlas-2018/), World Ocean Circulation Experiment (WOCE)-Argo Global Hydrographic Climatology (WAGHC^6,49^; https://www.cen.uni-hamburg.de/en/icdc/data/ocean/waghc.html) and SeaDataCloud (SDC; https://www.seadatanet.org/Products#) global climatology^7^.

The ANAS23 is relatively free from spikes, vertical instabilities, and artificial waters. In other words, it accurately reproduces the thermodynamic structures of the Kuroshio and its localized branches and spatial distribution of water masses. It also captures the thermohaline fronts and quasi-persistent mesoscale eddies. The approach proposed in this study has the potential to be applied to other marginal seas with large-scale marine ecosystems, presenting broad implications in the context of global warming. The resulting atlas can serve as an alternative climatology for regional studies.

## Methods

### Dataset of *in-situ* profiles

**Fig. 4:**
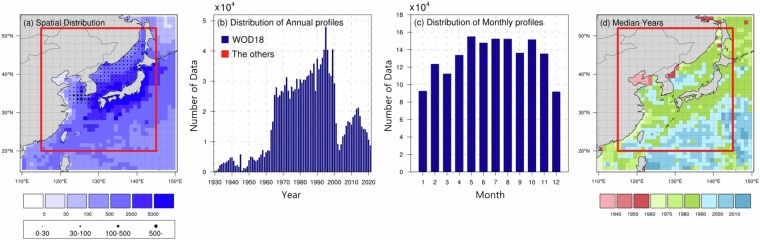
(**a**) Spatial distribution of the number of WOD18 (shaded), KIOST, and CREAMS (dots) within each 1° × 1° grid box. The distribution of (**b**) annual and (**c**) monthly profiles from WOD18 (blue bars), KIOST, and CREAMS (red bars). The distribution of annual profiles obtained before 1930 (1895–1929) is omitted from the time-series plot. (**d**) Spatial distribution of the median year of profiles within each 1° × 1° grid box. The ANAS23 domain (115°–145°E, 20°–52°N) is enclosed by the red box in (**a**) and (**d**).

ANAS23^43^ encompasses the marginal seas in the western North Pacific (115°–145°E, 20°–52°N). Given a data searching radius at the boundaries, this study collected *in-situ* hydrographic profiles occupied within the region of 110°–150°E and 15°–55°N over the period 1895−2021 (see Fig. 4 and Table 1). As a prime dataset, we extracted 1,587,422 temperature profiles and 833,216 salinity profiles from the World Ocean Database 2018 (WOD18^50^; https://www.ncei.noaa.gov/products/world-ocean-database). We only utilized data that passed the automated quality control checks recommended by the International Quality-Controlled Ocean Database (IQuOD) v0.1. These profiles were sourced from multiple instrumental platforms: Ocean Station Data (OSD), Conductivity–Temperature–Depth (CTD), Mechanical Bathythermograph (MBT), Expendable Bathythermograph (XBT), Moored Buoy (MRB), Argo Profiling Float (PFL) and Drifting Buoy (DRB). Autonomous Pinned Bathythermograph (APB), Surface-Only (SUR), and Glider (GLD) are excluded from the development because they are not appropriate for climatological estimation^7^. To manage a time-varying warm bias in the XBT and MBT profiles, we took advantage of the corrected ones by considering the observation period, water temperature, and instrument types^51^. There are additional 1,530 CTD profiles (0.1% of the total data used for the ANAS23 construction) that consist of data obtained by the Korea Institute of Ocean Science and Technology (KIOST; 10.22808/DATA-2025-1) and Circulation Research of the East Asian Marginal Seas (CREAMS; 10.6084/m9.figshare.5358058.v2) expedition^52,53^. These profiles span the period from 1981 to 2004 and are concentrated in the seas around the Korean Peninsula (Fig. 4b). The number of profiles used each month exceeded 90,000, with summer having the highest profiles at approximately 150,000, and winter having the lowest profiles at about 100,000 (Fig. 4c).

**Table 1:** The number of vertical profiles by instrument type and source.

Source	WOD18	KIOST	CREAMS	Measurement
Type				
OSD	536,350			T/S
CTD	155,170	916	614	T/S
MBT	352,055			T
XBT	326,837			T
MRB	73,612			T
PFL	141,696			T/S
DRB	1,702			T
Total	1,587,422	916	614	1,588,952

T and S indicate temperature and salinity, respectively.

The median year of observed profiles indicates the representative year of collected observation within a 1° × 1° grid box (Fig. 4d). Although the overall median year for this study area is in the mid-1980s, the spatial distribution of these observations is not uniform. Specifically, ship-based profile measurements were predominantly conducted prior to the early 2000s. However, ARGO-based observations have gradually replaced ship-based ones particularly in the open ocean since the early 2000s. Hence, the representative years for this region are relatively recent. There is a significant lack of data in the Bohai Sea and Wonsan Bay in the East Sea; most observations were even occupied before the 1950s. Such a scarcity of data could yield uncertainties that hinder the accuracy of the climatology, which will be discussed. To estimate the bottom topography and land-sea mask for the ANAS23, we used 1 arc-minute data from ETOPO2022, available at www.ncei.noaa.gov/products/etopo-global-relief-model.

### Dataset for the validation of ANAS23

We compared ANAS23 with WOA18, WAGHC, and SDC which have a quarter-degree horizontal resolution, as well as EAS-RC with a tenth-degree resolution. Here is a brief overview of these climatologies. The WOA18 adopted the Barnes’ objective interpolation scheme^54^, which uses a successive iteration to correct the first guess field. It also performed a three-pass correction with influence radii of 321, 267, and 214 km to avoid the formation of an artificial feature such as filament, eddy, and front^2^. This multi-iteration correction with large search radii mitigates noise, but oceanic features may be overly filtered. Furthermore, the fact that the Barnes approach is based on the correction of the first guess field suggests that the gridded product might be determined by the initial field, particularly where observations are sparse. The WOA18 is a compilation of six climatological datasets spanning decades from 1955 to 2017, with the central year of this collection being 1984^6^. This approach avoids weights toward data-abundant years, thus preventing spatial disparity.

Recently developed WAGHC provides two versions of the climatology, mapped on isobaric and isopycnal surfaces, using the optimal interpolation (OI) approach^55^ with a decorrelation scale length of 333 km^6^. For this study, we mainly used the isopycnal one, which is known to preserve the nonlinear seawater mixing process for seawaters, thus avoiding artificial hydrographic structures^56^. Since only WAGHC is interpolated on isopycnals and others on isobars, the comparison would reveal their differences depending on the mapping method. Besides, the WAGHC predominantly incorporates data obtained from 1985 to 2016, offering a more contemporary representation (median years of around 2010) of the mean state of the ocean in comparison to other gridded products^6^.

SDC version 1^57^ (SDC hereafter) was constructed using data spanning more than a century (1900–2017). Besides WOD’s quality control, the SDC implemented an additional quality control process, employing a repeated convergence approach by flagging two standard deviations as outliners from the mean value within a 5° by 5° horizontal grid. This method eliminates nearly 15% of total profiles^7^. Furthermore, the data interpolating variational analysis method^58^, which is equivalent to the OI basically, was applied for horizontal interpolation with the 300 km influence radius. This approach renders reduced sensitivity to the first guess field^7^. Another distinct feature of SDC is the incorporation of a constraint term in the interpolation algorithm. This term reduces mapping errors caused by data crossing land-sea interfaces such as peninsulas, thus allowing for the representation of distinct water masses separated by land clearly without mixing their properties.

The EAS-RC is a tenth-degree atlas that used the same interpolation method as WOA18, and applied a three-pass filter with influence radii of 211, 155, and 111 km. The EAS-RC is a climatology produced using observational data collected over several decades from 1804 to 2013^6^. The EAS-RC and ANAS23 were directly compared because they have the same horizontal resolution of 0.1° × 0.1°. Since WOA18, WAGHC and SDC provide a horizontal resolution of 0.25° × 0.25°, ANAS23 was regridded to a 0.25° × 0.25° horizontal resolution for their comparison.

For further validation of ANAS23, we employed hydrographic observation sections: the Japan Meteorological Agency 137°E (JMA137^59^) and Korea Oceanic Data Center 105 (KODC105^60^) lines. The JMA137 covers the western North Pacific, encompassing the Kuroshio Current with North Pacific Intermediate and Mode Waters^61^. On the other hand, the KODC105 traverses the East Sea along 37.6°N, which includes the Ulleung Warm Eddy and East Sea Intermediate Water^11^. Our study averaged the JMA observations at each station from 1967 to 2021 for January and the KODC observations from 1994 to 2021 for February, respectively. Additionally, surface geostrophic velocity data with a resolution of 0.25° × 0.25° and spanning 1993–2020 were obtained from the Archiving, Validation, and Interpretation of Satellite Oceanographic data (AVISO^62^) and used to assess the reliability of the geostrophic circulation field estimated by ANAS23.

### Procedure

To handle the historical data obtained from various sources, we employed a systematic procedure that incorporates unified quality control, vertical interpolation, horizontal kriging, smoothing, and vertical stabilizing. For a total of 1,588,952 temperature profiles and 833,216 salinity profiles, as mentioned above, we performed the following procedure in sequence. Note that we calculated monthly mean fields for depths ranging from 0 to 1,500 m, while we computed an annual mean field for the deeper layer ( > 1,500 m) since water properties in deep water exhibit marginal seasonality. The flowchart depicted in Fig. 1 provides an overview of the comprehensive production process employed to construct ANAS23.

#### Individual quality control of in-situ data

The range check decides whether data on observed levels exceed pre-specified values with respect to depth. Next, a gradient check removes data with excessive vertical gradient(s) in a profile. Herein, the vertical gradient of temperature (salinity) [$\varDelta T(S)/\varDelta Z$] was calculated for two adjacent layers, and the threshold values are shown in Fig. 1. Any profiles that exceeded the threshold were flagged and not considered for the construction of ANAS23.

#### Vertical interpolation to standard depth levels

The data points that were not flagged were subjected to vertical interpolation onto 73 standard depth levels. These standard depth levels were defined as follows: 0–100 m at intervals of 5 m, 125–500 m at intervals of 25 m, 550–1,500 m at intervals of 50 m, and 1,750–5,500 m at intervals of 250 m. The upper ocean has a higher vertical resolution, allowing for a more accurate representation of hydrographic states, including the thermocline layer.

We utilized the Gibbs Seawater oceanographic toolbox of the Thermodynamic Equation of Seawater-2010 (TEOS-10)^63^ for vertical interpolation. Specifically, we used the MRST-PCHIP method (function gsw_SA_CT_interp) for profiles containing both temperature and salinity, and the MR-PCHIP method (function gsw_t_interp) for profiles with temperature only. These interpolation methods are based on the Piecewise Cubic Hermite Interpolating Polynomial (PCHIP^64^) and demonstrated superior performance compared to other vertical interpolation schemes, such as linear interpolation, spline interpolation and the Reiniger and Ross’s approach^65^. The two algorithms using PCHIP require at least four data points for vertical interpolation. In cases where only two or three data points were available in an individual profile, we employed linear interpolation. If a profile contained only one data point that corresponded to a standard depth level, we used that value directly.

To ensure the stability of each vertically interpolated profile that recorded both temperature and salinity, we assessed the density difference between two adjacent layers [${\rho }_{i+1}-{\rho }_{i}$, where $i$ denotes the standard depth level ranging from 1 to 72]. If the density difference was less than 10^−3^ kg·m^−3^, we considered the water column potentially unstable; in such cases, we flagged the two corresponding data points. Furthermore, we discarded a profile that rendered two or more density inversions.

#### Quality controls at a standard depth level

Unlike the previous steps for individual profiles, the current phase of quality control was executed on collective data at an identical depth. We performed horizontal quality control by applying a one-time 3-standard deviation (σ) check. Data outside 3-σ from the mean within a 1° × 1° grid are flagged for the upper 1,500 m. For the depths deeper than 1,500 m, characterized by minimal seasonal fluctuations, a broader 3° × 3° grid was employed. This process flagged 1.25% of the total data.

#### Horizontal interpolation

We conducted horizontal interpolation for 0.1° gridded monthly mean fields from 0 to 1,500 m using carefully quality-controlled data. Typically, spatial prediction algorithms, like the optimal interpolation method, calculate weights considering only distance, using inverse distance weighting with a fixed influence radius. Our study employs the simple kriging method, which relies on variograms to quantify spatial variability among adjacent data points and incorporates this information into objective estimates to minimize errors^66–68^. The weight is determined to minimize error variance between predicted and true values by considering several factors. These include the spatial continuity of observation data, the correlation between grid locations and neighboring observation data, and the spatial correlation of observation data through the variogram, which allows the influence radius to vary dynamically. Kriging interpolation has been widely used for gridding climate variables due to its ability to consider spatial correlations among observations without needing a first-guess field. However, this method entails estimating a variogram at each grid point, meaning this method is computationally intensive and unsuitable for applications on a global scale, such as creating a global atlas.

This study’s default for the influence radius was set to 45 km. The maximum number of observed data within the influence radius was set to 80, and the minimum number of observed data within the influence radius was set to 5. Therefore, when the number of observed data within the influence radius of 45 km is selected between 5 and 80, the selected observed data are used to compute the objective estimates. Also, if the number of observed data within the influence radius of 45 km exceeds 80, the influence radius will be reduced until it becomes equal to the maximum number of observed data. On the other hand, if the number of observed data within the influence radius falls below 5, the influence radius will be increased until it becomes equal to the minimum number of observed data. Therefore, the influence radius varies according to the distribution of observed data. The influence radius set in this study is the smallest compared to the influence radius set by other climatologies (*i.e*., 211, 155, and 111 km for EAS-RC; 321, 267, and 214 km for WOA18; 333 km for WAGHC; and 300 km for SDC). We have concluded that applying a relatively small radius is more appropriate than using a large radius applied in other climatologies^10,12,14^, when considering the spatial and temporal scales of the ocean environment in the East Asian Seas^31,69^.

Additionally, the horizontal interpolated grid data using the kriging method were processed through two filters to remove noise signals. One of the two filters used is the Barnes filter. The Barnes filter is a useful tool for spatial interpolation and smoothing of observation data. It assigns weights to surrounding observation data based on their distances from grid points within the influence radius and performs the filtering process. The applied influence radii for the Barnes filter are 211 km, 155 km, and 111 km, the same as those used in EAS-RC. The other one is the nine-point smoothing filter. The nine-point smoothing filter is a technique that involves updating the value of a grid point by incorporating 75% of its original value and 25% of the values from its surrounding eight grid points.

#### Vertical correction

Unstable water mass, which primarily results from abnormal data or individual mapping for temperature and salinity, is regarded as an undesirable characteristic in long-term mean climatology^2,5^. We attempted to correct the problematic vertical structure using a numerical algorithm in the TEOS-10^44^. The algorithm minimally adjusts the hydrographic data for the density correction. The correction process was performed on the entire layers after merging the monthly climatology for depths of 0–1,500 m and annual one from 1,750 m to the bottom.

## Data Records

The dataset is available at the KIOST repository (10.22808/DATA-2024-1; https://sciwatch.kiost.ac.kr/handle/2020.kiost/45363)^43^, it has been assigned an open license (CC-BY). This dataset is provided in NetCDF format (ANAS23t_mon_ver1.nc) and comprises four main variables: temperature (abbreviated as tmp), salinity (sal), and their error variances (tmp_errvar and sal_errvar). The dataset has a horizontal resolution of 0.1° × 0.1°, covering from 20°N to 52°N (320 grids) and from 115°E to 145°E (300 grids); the depth is divided into 73 layers from the surface to 5,500 m.

## Technical Validation

### Horizontal distribution of temperature and salinity

**Fig. 5:**
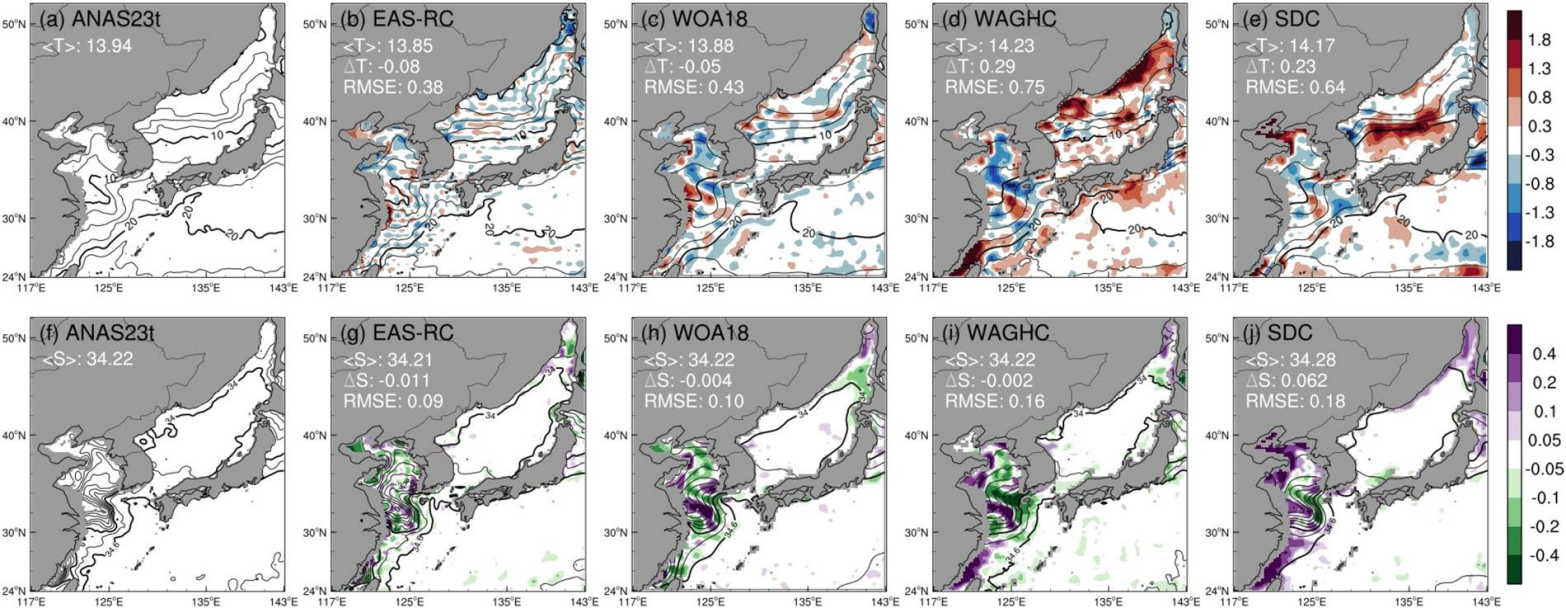
Horizontal distribution of temperature (2.5 °C contour interval) and salinity (0.3 contour interval) and the difference (shaded) with respect to the ANAS23 at 20 m in winter (Jan–Feb–Mar). The upper (lower) figures are for the temperature (salinity); the top left corner in each figure shows the area average, its difference, and root mean square error (RMSE) between each climatology and the ANAS23. The isotherms (isolines) of 0, 10, and 20°C (34.0 and 34.6) are shown in bold.

We initiated a validation process for ANAS23’s performance, focusing on the winter (Jan–Feb–Mar) mean horizontal distribution of temperature and salinity at a surface layer (Fig. 5). We compared ANAS23 with EAS-RC, WOA18, WAGHC, and SDC, examining their deviations. Spatially, the mean temperature difference between ANAS23 and EAS-RC was calculated to be -0.08°C, with a root mean square error (RMSE) of 0.38°C, confirming that the temperature distribution of the two datasets is mostly similar. However, the temperature deviation distribution of EAS-RC showed a grid pattern distribution in the East Sea, Yellow Sea, and East China Sea (Fig. 5b). This abnormal temperature error pattern observed in EAS-RC was also mentioned in previous research^70^.

In the Yellow Sea, ANAS23 and EAS-RC showed similar distributions, except for the Yangtze River outflow area. A distinctly warm and saline tongue-like feature is more prominent in ANAS23 and EAS-RC than in the quarter-degree resolution climatologies (*i.e*., WOA18, WAGHC, and SDC), indicative of the pronounced flow of the Yellow Sea Warm Current^31,71–74^ (Fig. 5). Compared to other climatologies, the ANAS23 showed warmer and saltier spatial distribution along the Yellow Sea Warm Current and colder and fresher spatial distribution along the China Coastal Current^14,73^. These anomalies constitute the S-shape frontal structure, underscoring that the ANAS23 seems to reproduce the distinct thermohaline front and enhanced anticyclonic circulation over the Yellow Sea compared to EAS-RC, WOA18, WAGHC, and SDC. This distribution of temperature and salinity aligns with the characteristic hydrographic structure during the winter monsoon period^72^ (see Fig. 5).

The WOA18 showed similar average temperature ( < T >  = 13.88°C) and salinity ( < S >  = 34.22), and RMSE (0.43°C for temperature and 0.10 for salinity) to EAS-RC (Figs. 5c and 5h). This indicates spatial similarity despite the difference in spatial resolution between WOA18 and EAS-RC. However, in the Yellow Sea, climatologies with quarter-degree resolution (WAGHC and SDC) including WOA18 showed low temperature and low salinity, which may lead to weakening of the Yellow Sea Warm Current.

The WAGHC showed surface warming (Fig. 5d) overall in areas except the Yellow Sea. This is presumably because it was produced using more recent observational data compared to other climatologies. Notably, the waters south of Japan exhibit an anomalous warming band, with temperatures up to 1°C higher than in ANAS23. It is likely in line with the northward migration of the Kuroshio’s primary axis over the preceding four decades^33,75^. Furthermore, the WAGHC exhibits prominent warm biases along the coastlines of Siberian and North Korea, which can be attributed to the influence of the Liman and North Korea Cold Currents within the subpolar gyre of the East Sea. These anomalies, at least in part, result from WAGHC incorporating more recent observational data, particularly pertinent to Argo profiles.

The SDC showed strong positive temperature anomalies exceeding 1.8°C in the polar front of the East Sea along 39.5°N (Fig. 5e). The salinity distribution of SDC showed significant discrepancies in the Tatar Strait, Bohai Sea, and Taiwan Strait (Fig. 5j). These discrepancies could be attributed to limited historical observations in these regions, which were collected in the distant past (see Fig. 4c). This paucity of data could hinder the accurate estimation of climatological mean values for these specific areas.

**Fig. 6:**
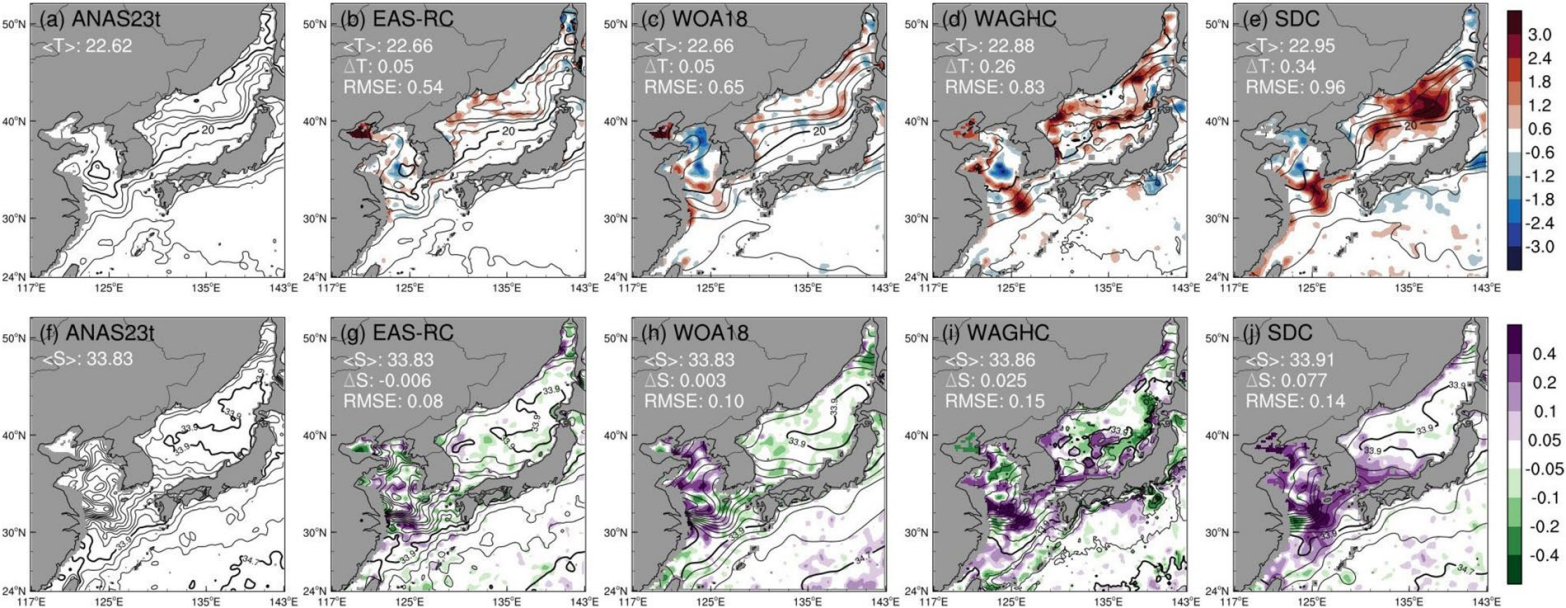
The same as Fig. 5 except for summer (Jul–Aug–Sep). Contour intervals are 2 °C and 0.2 psu, respectively. The isotherms (isolines) of 10 and 20 °C (33.9 and 34.7) are shown in bold.

For summer, each climatology showed spatial differences in the East Sea, Yellow Sea, and East China Sea (Fig. 6). The EAS-RC provides the most similar result to ANAS23 in terms of the area average (<T> = 22.66°C and <S> = 33.83) and RMSE (0.54°C for temperature and 0.08 for salinity). However, the EAS-RC exhibited a strong warm bias in the Bohai Sea compared to other climatologies (Fig. 6b). For the quarter-degree resolution, WOA18 showed a similar spatial distribution to ANAS23 and EAS-RC in terms of spatial average and RMSE when compared to WAGHC and SDC (Figs. 5c and 5h). The SDC depicts the warmest East Sea among the four climatologies, showing a maximum deviation that exceeds 3°C over the subpolar area. This warm bias in the East Sea is mainly responsible for the largest mean difference and RMSE between SDC and ANAS23 (0.34°C and 0.96°C, respectively). WAGHC and SDC also estimate fairly high temperature (>2.4°C) and salinity (>0.4) biases around Jeju Island, which might lead to an enhancement of the Jeju Warm Current^73^.

### JMA137: the meridional section south of Japan along the 137°E

**Fig. 7:**
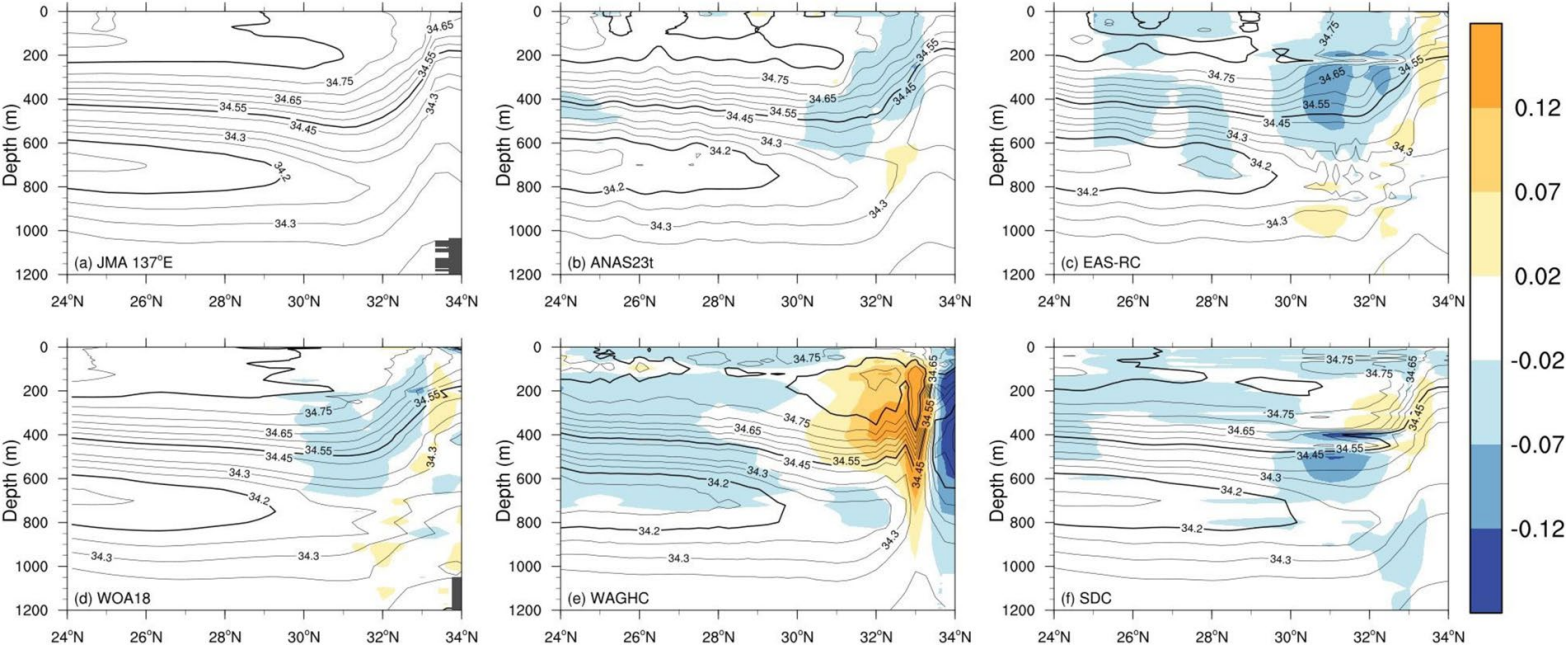
Vertical section of salinity (0.05 contour interval) and the difference (shaded) with respect to the JMA observation line along 137°E in January.

Next, we validated ANAS23’s gridded vertical temperature and salinity through a comparative analysis with repeated hydrographic observations. Our validation begins with an assessment of January salinity observed from the JMA137 section (Fig. 7), averaged over the period 1967–2021. The Kuroshio current generally intersects the section near 33°N while occasionally meandering offshore up to 30°N^61^. The long-term mean temperature and salinity distribution, complemented by acoustic Doppler current profiler observations, reveals the presence of a counter-coastal current to the north of the Kuroshio^61^. The major water masses observed in this section include NPIW, which is centered around 700 m and 24°N latitude at this longitude. Besides, the Subtropical Mode Water (STMW) can be identified by its uniform subsurface water with a temperature range of 16°C to 19°C south of the Kuroshio^61,76–78^.

While ANAS23 exhibits comparable or marginally lower salinity than the JMA137 observation (Fig. 7b), the EAS-RC showed extensive and robust negative anomalies within the upper waters above the NPIW (Fig. 7c). In the EAS-RC, the artificial, noise-like pattern between 31°N and 33.5°N is prominent within the NPIW layer between 600 m and 1,000 m but not in the JMA137 observation and ANAS23 (Fig. 7). This perturbation might be due to that the data are gridded separately at a horizontal level, thus potentially containing density inversions^6^. To address the instability issue in a gridded profile, the EAS-RC adjusts temperature or salinity based on the sign of the vertical gradient in water properties^2,44,79^. When unstable data occur, vertical gradients of temperature and salinity are estimated; the temperature was preferentially adjusted to stabilize the profile when the temperature gradient was positive (i.e., warmer waters were located below). If the temperature gradient was normal and the salinity gradient was negative (i.e., fresher water below), the salinity was then adjusted. The problem is that the negative salinity gradient is common in the layer above a salinity-minimum water mass. Therefore, if the salinity profile is adjusted to have a positive gradient for this layer, this stabilization process may render an artificial water mass and result in such a wiggled perturbation. Another candidate for the perturbation could be the vertical interpolation method. The Reiniger and Ross’s vertical interpolation scheme^65^, which the EAS-RC has adopted, can generate overshoots in the interpolated profile when applied to water columns with vertical reversal(s) – in this case, intermediate water with salinity reversal^63^. Although its verification is beyond the scope of this study, this speculation suggests that a gridded profile in vertical interpolation and stabilizing procedures should be treated with caution^5,12,63^. The WOA18 was generated using the same method as the EAS-RC but showed a different spatial distribution. In particular, WOA18 showed a similar spatial pattern to observations despite its lower resolution compared to the EAS-RC (Fig. 7d). In WOA18, the sawtooth pattern between 31°N and 33.5°N is prominent within the NPIW layer between 600 m and 1,100 m. This sawtooth pattern is an abnormal phenomenon caused by unstable data during vertical profile interpolation, suggesting that it can lead to an abnormal geostrophic current.

The WAGHC shows extensive and robust negative anomalies within the upper waters above the NPIW. These anomalies are consistent with the horizontal distribution of surface salinity in the open ocean, as illustrated in Fig. 5i, attributed to more recent data for developing WAGHC^80^. In contrast to the negative anomalies, the WAGHC shows remarkably positive discrepancies exceeding 0.12 from the observation around south of 33°N, in alignment with the northward migration of the Kuroshio^75,81^, which is the core of warm and saline water from the tropical Pacific. Besides, N-shape spikes are evident within the positive anomalies around 33°N (Fig. 7e). These spikes represent unwanted noise, potentially contributing to the formation of artificial water masses along the main axis of the Kuroshio. We confirmed that the isobarically interpolated version of WAGHC has a similar pattern, albeit slightly reduced anomalies of up to 0.1 within Kuroshio’s main axis at 300 m depth. These observations imply that the isopycnal version of WAGHC does not outperform the isobarically interpolated products, at least in the vicinity of the Kuroshio, suggesting that the mapping along an isopycnal may not be effective in accurately reproducing a thermodynamic front or a newly generated water mass in the regions where the isopycnals emerge at the surface^6,82^.

**Fig. 8:**
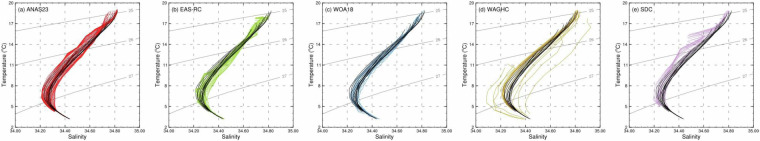
Temperature–salinity diagrams at 137°E in January. The domain for the diagrams is [30–34°N, 200–1000 m]. The JMA 137°E hydrographic observation data are superimposed in black lines. The grey lines indicate isopycnals of 25.0, 26.0, and 27.0 kg/m^3^.

We further investigate the hydrographic characteristics using a temperature–salinity diagram for the JMA137 section (Fig. 8). The diagram illustrates the salinity minimum layer (i.e., the NPIW) and its core with a temperature between 6° and 8°C (black lines in Fig. 8). For ANAS23, negative discrepancies in salinity from the observations are found within the upper ocean (Fig. 8a). The EAS-RC and WOA18 have temperature and salinity inversions in the water layer where the potential density is lower than 27 kg/m^3^ (Figs. 8b and 8c). Therefore, vertical interpolation and stabilizing procedures must be handled carefully when producing gridded profiles. The WAGHC’s salinity has significant deviations in and around the Kuroshio current, as confirmed in Fig. 7e, and the same is true for the temperature (Fig. 8d). This implies the presence of anomalously warm and saline water to the south of the Kuroshio, while cold and fresh water in the northern region only in WAGHC, highlighting again that the mapping on isopycnal surfaces does not always prevent the creation of artificial water masses.

The SDC contains marked salinity reversals in the water layer with a potential density of lower than 26 kg/m^3^ (Fig. 8e). This questionable vertical structure implies the formation of unstable water masses, which should be rectified by rigorous quality control or stabilization procedures. Notably, the SDC contains a significant proportion of unstable water columns, accounting for 58.2% and 23.0% of the surface ocean grid points (8,832) in February and August, respectively. Here, a gridded vertical profile is considered unstable when the buoyancy frequency (or Brunt-Väisälä frequency) squared is less than 1.5 × 10^−7^ s^−2^ at any layer^12,79^. Also, WAGHC exhibits unstable grid points (8.7%) for the February gridded field, particularly in regions along the Kuroshio and of limited data availability.

### KODC105 line: the zonal section in the southwest East Sea along 37.6°N

**Fig. 9:**
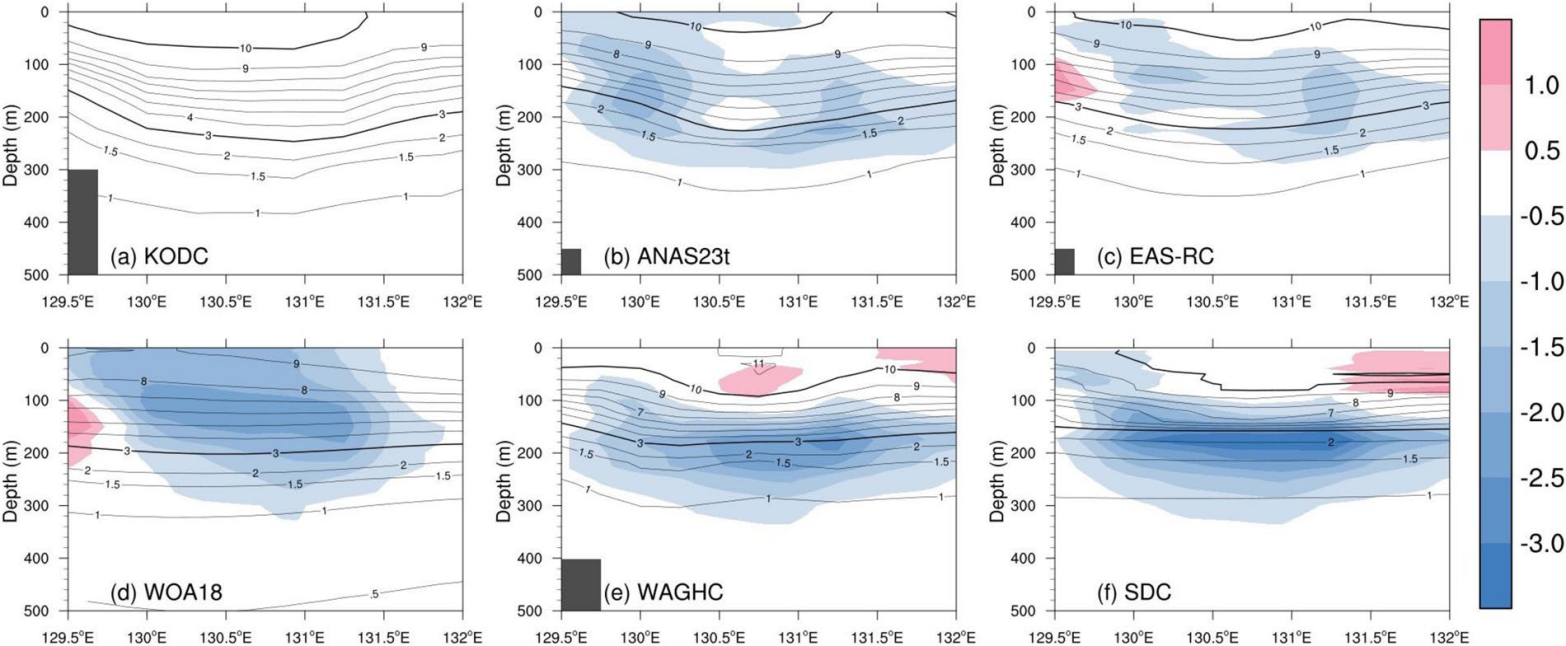
Vertical section of temperature (contour; °C) and the difference (shaded) with respect to the KODC observation line along 37.6°N in February.

Figure 9 depicts the vertical temperature observed along the 37°N in the southwest East Sea (KODC 105 line). The CTD casts occupied during February are averaged from 1994 to 2021, representing the mean temperature for the year 2008. The observed temperature exhibits a distinct concave pattern within the warm subsurface layer above 1.5 °C (Fig. 9a). This signal reaches up to 300 m and indicates a quasi-persistent anticyclonic eddy, i.e., Ulleung Warm Eddy (UWE). This eddy is mainly fueled by the East Korea Warm Current, a northward branch of the Tsushima Warm Current^37,83^ (see Fig. 2). Volume transport across the Korea Strait has increased persistently since the late 1970s^84^. As a result, the UWE is expected to warm and spin up, consistent with the highest sea level rise rate of more than 7 mm/yr from 1993 to 2019 around the seas of Korea^85^. To the west of the UWE, the East Sea Intermediate Water (ESIW), a subsurface salinity minimum layer (<34.06) with low temperature (1°C–5 °C) as a part of the subpolar gyre in the East Sea, flows from north to south along the east coast of Korea^86–88^. The core of the ESIW is located around 100 m on the eastern coast of Korea at this latitude.

Figure 9 reveals noticeable cold anomalies compared to the observations, particularly at the subsurface center of the UWE. These substantial anomalies can be attributed to the fact that the observations are averaged over the past 27 years, reflecting robust recent warming over the Ulleung Basin. The ANAS23 showed expected spatial patterns compared to observations including physical features of UWE and ESIW (Fig. 9b). However, the EAS-RC does not properly reproduce the characteristics of the ESIW: the ESIW has up to 1.7°C warmer and 0.09 saltier properties than observed (Fig. 9c). Noteworthy is that the WOA18 fails to accurately capture the concave structure of not only ESIW but also UWE, despite using similar procedures and data as the EAS-RC (Fig. 9d). This indicates structural differences due to the spatial resolution between WOA18 and EAS-RC. This observation demonstrates that the bias, particularly over the continental slope, is attributed to the large influence radius employed for horizontal interpolation. If gridded values are calculated using a large influence radius near the continental slope where topography changes drastically, more exterior ocean data could be used. As a result, a climatology with large influence radius tends to forge warmer and saltier water masses than the observation on the slope along boundary current^12^.

The WAGHC and SDC depict the features of the UWE near the surface layer, but also substantial cold biases at 100 to 300 m, along with warm bias at the surface layer (Fig. 8e and f), thereby resulting in enhanced stratification than the observation.

### The East China Sea to the North Pacific: the zonal section along 30°N

**Fig. 10:**
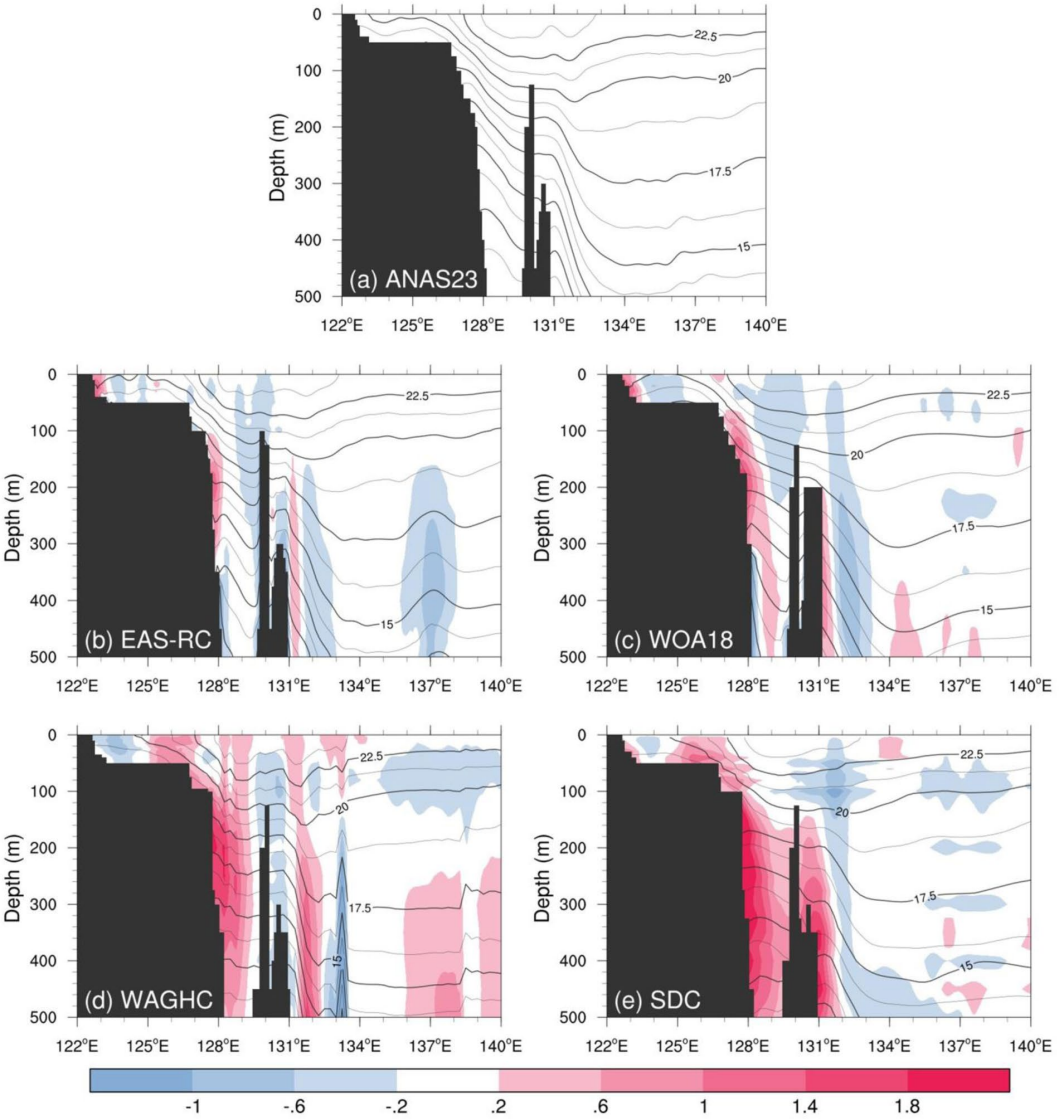
Vertical section of annual mean temperature (contour; °C) and the difference (shaded) with respect to the ANAS23 along 30°N.

We now examine the vertical section of annual mean temperature along 30°N (Fig. 10), where the Kuroshio and complex topography are crucial in shaping the hydrographic properties^12^. This section shows two current cores: the Kuroshio flows northwestward along the 200 m isobath within the Okinawa Trough until approximately 30°N and meanders southward to exit through the Tokara Strait, thereby showing another current core approximately at 131.5°E^34^.

The differences in the temperature patterns are obvious throughout the domain but are particularly considerable below the subsurface along the continental slope. The EAS-RC and WOA18 exhibit a positive temperature bias in front of the Yangtze River, forming a dome-like thermal structure over the East China Sea (Fig. 10b and c). This bias likely leads to an anomalous southward current across the Taiwan Warm Current. Additionally, the isothermal slope of EAS-RC depicts a meandering shape along the continental slope. These isothermal slopes can cause unrealistic geostrophic currents. In particular, the cold anomalies around 137°E could lead to unrealistic geostrophic currents related to the Kuroshio Current and recirculation.

The quarter-degree climatologies also yield substantial warm anomalies in the Okinawa trough compared to ANAS23’s temperature distribution. These warm biases, associated with the large influence radius for the global atlas, lead to unrealistic isothermal slopes in the trough, which could result in a weaker western boundary current or a localized counter current. Besides this large-scale bias, there are also minor but noteworthy error-like features in the WAGHC. Wedge-shaped noise approximately is observed around 133°E; this cold anomaly is bottom intensified and reaches the upper thermocline (Fig. 10d). Another irregularity is observed near 138.5°E, where isothermal lines abruptly rise toward the open ocean, reflecting unrealistic recirculation of the Kuroshio.

### Geostrophic current

**Fig. 11:**
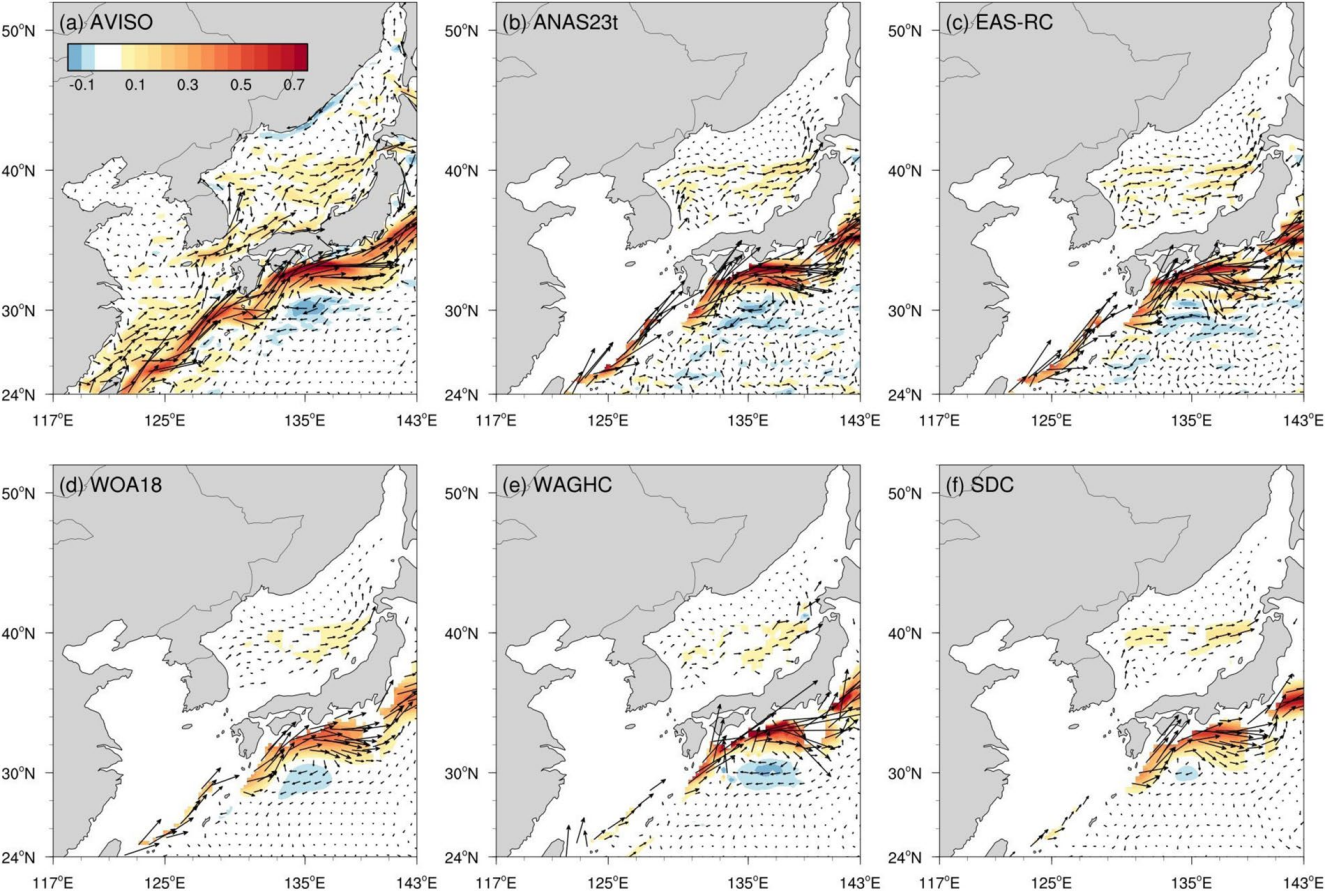
Annual mean geostrophic current (vector; m/s) and the zonal component (shaded) from the (**a**) AVISO, (**b**) ANAS23, (**c**) EAS-RC, (**d**) WOA18, (**e**) WAGHC, and (**f**) SDC. In (**b**)–(**f**), the velocity relative to 1,000 dbar is calculated from the temperature–salinity field.

Figure 11 shows the annual mean geostrophic current in the marginal seas in the western North Pacific estimated from merged satellite altimetry data averaged from 1993 to 2022 and from annually averaged temperature and salinity fields of each climatology relative to 1,000 dbar. The five climatologies reproduce the current system well compared to that from the satellite altimetry data. The most noticeable current in the observation is the Kuroshio and the Tsushima Warm Current flowing through the Korean Strait and its branches in the East Sea (Fig. 11a). The Kuroshio mainly enters the east of Taiwan to flow along the Okinawa Trough, and part of the current intrudes into the Luzon Strait. The zonal speed of Kuroshio’s core at 137°E section is 0.59 m/s for the satellite altimetry data, and ANAS23 is 0.56 m/s, which is most similar to the observation among the five climatologies (EAS-RC 0.44 m/s, WOA18 0.42 m/s, WAGHC 0.74 m/s, and SDC 0.46 m/s). As mentioned, while WAGHC and SDC show relatively weak currents at the Okinawa Trough, ANAS23 renders the strongest northeastward current core.

Our comparison, based on water mass properties and oceanic circulation, shows that the ANAS23 depicts well the overall hydrographic characteristics of the marginal seas in the Northwest Pacific. Previous studies have demonstrated that anthropogenic activities significantly impact water temperature and salinity, which is expected to increase climate variability^80,89,90^. In this regard, we hope that our study, including the gridded product ANAS23, can contribute to a better understanding of the physical oceanography of the Northeast Asian seas, particularly in the context of ongoing climate change by using it as an initial and boundary conditions for a numerical experiment.

## Data Availability

All processing, including quality control (QC) processes for all observational data used to produce ANAS23^43^, was conducted using MATLAB (version R2022a). The simple kriging method (The Kriging Toolbox version 3.0) used for horizontal interpolation utilized the toolbox provided by MATLAB (https://globec.whoi.edu/software/kriging/V3/intro_v3.html). Vertical interpolation and stabilization were carried out using the Gibbs Seawater oceanographic toolbox of the TEOS-10 (https://github.com/TEOS-10/GSW-Matlab), as mentioned in the Methods section. Nine-point smoothing utilized a function inherent in NCL (NCAR Command Language, https://www.ncl.ucar.edu/Document/Functions/Built-in/smth9.shtml). The customized MATLAB and NCL scripts and exampled dataset, ANAS23_script.zip, can be downloadable from the KIOST repository (https://sciwatch.kiost.ac.kr/handle/2020.kiost/45363/).
